# Versatility of the Templated Surface Assembly of Nanoparticles from Water-in-Oil Microemulsions in Equivalent Hybrid Nanostructured Films

**DOI:** 10.3390/nano14211726

**Published:** 2024-10-29

**Authors:** Rebeca Fortes Martín, Sibylle Rüstig, Ilko Bald, Joachim Koetz

**Affiliations:** Institute of Chemistry, Universität Potsdam, 14469 Potsdam, Germanybald@uni-potsdam.de (I.B.)

**Keywords:** electron microscopy, interfacial assembly, microemulsions, nanoparticles, nanostructures, self-assembly, Winsor phases

## Abstract

Water-in-oil microemulsions, as stable colloidal dispersions from quasi-ternary mixtures, have been used in diverse applications, including nanoreactors for confined chemical processes. Their use as soft templates not only includes nanomaterial synthesis but also the interfacial assembly of nanoparticles in hybrid nanostructures. Especially the hierarchical arrangement of different types of nanoparticles over a surface in filament networks constitutes an interesting bottom-up strategy for facile and tunable film coating. Herein, we demonstrate the versatility of this surface assembly from microemulsion dispersions. Transmission and Scanning Electron Microscopy, in addition to UV–Vis Transmittance Spectroscopy, proved the assembly tunability after solvent evaporation under different conditions: the nanostructured films can be formed over different surfaces, using different compositions of liquid phases, as well as with the incorporation of different nanoparticle materials while keeping equivalent surface functionalization. This offers the possibility of adapting different components and conditions for coating tuning on a larger scale with simple procedures.

## 1. Introduction

The assembly of nanoparticles in ordered structures represents a suitable bottom-up approach for the production of functional materials [1,2,3,4,5]. Various optoelectronic applications of nanoparticle assemblies include, for instance, the fabrication of new nanomaterials for catalysis, sensing, and nanoelectronics [1,2,3,6,7,8,9,10]. Additionally, the incorporation of different nanoparticles in hybrid nanostructures enables the combination of collective properties and functionalities [6,11,12].

To form ordered assemblies, the interactions between nanoparticles should be controlled. These interactions can be attractive or repulsive and can involve intermolecular, electrostatic, dipolar, and magnetic forces [3,10,13,14,15,16]. Additionally, the nanoparticle interactions may be intrinsic to the nanoparticle composition or provided by functionalization at the nanoparticle surface, and can be triggered by changing the medium conditions, incorporation of additives, applying an external field, or solvent evaporation [1,2,3,5,8,10,11,14,15,16]. Nanoparticle assemblies can provide colloidal superstructures distributed within a certain space [2,6,8,9,11,17,18], or ordered arrangements over a surface as arrays, close-packed monolayers, chains, or related thin films [1,2,3,6,7,8,9,10,11]. Furthermore, the use of templates can afford hierarchical assemblies in anisotropic shapes. A hard template typically consists of a solid scaffold with pre-defined surface patterns, while a soft template phase includes self-organized dispersions of polymer structures or segregated liquid phases [1,2,4,6,7,9,14,15,17,18]. Especially the interfacial assembly can direct interactions between nanoparticles from different liquid phases and contributes to form interconnected channels, encapsulations, or other organized nanocomposites [2,9,17,18,19].

Microemulsions consist of optically clear liquid-in-liquid dispersions that are thermodynamically stable by surface active agents. According to the distribution of the liquids as dispersed and continuous media, they can be classified as oil-in-water microemulsions (also named normal microemulsion phase), as water-in-oil microemulsions (also named reverse microemulsion phase), or as interconnected liquid phases in bicontinuous microemulsions [20,21,22,23]. The applications of microemulsions can involve multiple processes in pharmaceutical, catalytic, cosmetic, and coating applications [21,23]. For instance, water-in-oil microemulsions have been extensively studied in recent decades as soft templates in nanoparticle synthesis [22,23,24,25].

Recently, microemulsions were also proven to be suitable as a soft template for nanoparticle assembly at liquid–liquid interfaces [26,27,28,29,30,31]. In this case, it was possible to combine different types of nanoparticles of different liquid dispersibility according to their surface functionalization. Their interfacial assembly in water-in-oil microemulsions involved different nanomaterial compositions, dispersed in either oil or water. Thus, oil-dispersible, superparamagnetic iron oxide nanoparticles (SPIO(Ol)NPs) were dispersed in the continuous oil phase and water-dispersible, polyethylenimine-coated gold nanoparticles (Au(PEI)NPs) were incorporated within the water droplets. Especially the control of nanoparticle aggregation can be useful for obtaining ordered assemblies with defined patterns [26,28,29]. For the surface assembly of nanoparticles in filament networks by drop-casting of an upper reverse microemulsion phase [28,29], two main processes were interpreted. On the one hand, the formation of nanoparticle heteroclusters by restricted aggregation in an upper microemulsion phase was attributed to the electrostatic charge suppression of the Au(PEI)NPs with the sulfonate groups of 2-ethyl-hexyl sodium sulfosuccinate as surfactant, referred to as Aerosol-OT (AOT). On the other hand, the elongation of microemulsion droplets should also contribute to the elongated nanostructures after solvent evaporation [28]. Thus, these interfacial processes enable the formation of filament networks over the microscale from spherical nanoparticles smaller than 10 nm.

In this study, the versatility of reverse AOT microemulsions as self-organized dispersions, to mediate the interfacial assembly of nanoparticles in nanostructured films, is demonstrated. Firstly, the resulting nanostructures can be observed over different surfaces and different deposition conditions, using different electron microscopy techniques. Additionally, the use of different organic solvents in the oil phase can be of relevance for the tunability of the structural organization of these surface assemblies. Moreover, the use of different nanoparticle compositions while keeping an equivalent surface functionalization was also suitable for the formation of these nanostructures. This opens possibilities of using different nanoparticle combinations for new fields of directed applications.

## 2. Materials and Methods

### 2.1. Samples Preparation

The following chemical reagents were purchased from commercial sources to use without further purification: dioctyl sodium sulfosuccinate (AOT), iron (III) acetylacetonate –Fe(acac)_3_–, gold chloride hydrate –HAuCl_4_∙3H_2_O–, and oleylamine were obtained from Sigma-Aldrich (St. Louis, MO, USA); oleic acid was obtained from AppliChem (Darmstadt, Germany); diphenylether from Alfa Aesar (Haverhill, MA, USA); cadmium chloride –CdCl_2_– and ammonia sulfide –(NH_4_)_2_S– from Fluka (Buchs, Switzerland); 1,2-hexadecanediol from TCI (Tokio, Japan); *n*-hexane and silver nitrate –AgNO_3_– from Roth (Karlsruhe, Germany), and *n*-heptane and 1-pentanol from VWR Chemicals (Radnor, PA, USA). *N,N’*-dimethyl formamide (DMF) and ethanol were technical-grade solvents. The hyperbranched polyethyleneimine (PEI) with a molar mass of 25,000 g/mol was obtained as Lupasol G100 from BASF (Ludwigshafen, Germany). Additionally, water was taken from a Millipore A+ Reference Milli-Q system.

The oleyl-capped nanoparticles were prepared by microwave-assisted syntheses in a Discover SP microwave reactor (CEM, Matthews, NC, USA). For the adapted synthesis of iron oxide nanoparticles –SPIO(Ol)NPs– [26,28,29,32,33]: Fe(acac)_3_ (0.2 mmol), hexadecanediol (1 mmol), oleic acid (0.6 mmol), and oleylamine (0.9 mmol) were mixed in pre-warmed diphenyl ether (2 mL) under magnetic stirring and in two heating steps at 2.9 bar with 800 W maximum power and 2450 MHz microwaves, first at 200 °C for 30 min and then heating at 265 °C for another 30 min. For the adapted synthesis of silver nanoparticles –Ag(Ol)NPs– [26,34,35]: AgNO_3_ (0.145 mmol) was dissolved in DMF (1 mL) for 30 min, before adding a mixture of oleic acid and oleylamine (1.16 mmol each), and the mixture was heated under magnetic stirring in two microwave-assisted steps, first at 110 °C with 100 W power for 30 min and then at 147 °C with 200 W for 5 min. The resulting dark mixtures were cooled down and then flocculated at room temperature with ethanol. The separation of the dark precipitates from the liquid supernatant was assisted either with an external magnet for the magnetic iron oxide nanoparticles or by centrifugation (6000 rpm, 15 min) for the non-magnetic silver nanoparticles. The resulting solids were re-dispersed in heptane with partial oleic acid and oleylamine, repeating these washing steps with ethanol and heptane, and the obtained nanoparticles were finally dried in a vacuum oven at 35 °C for about two days. The resulting SPIO(Ol)NPs (Figure S1) and Ag(Ol)NPs (Figure S2) form optically clear dispersions, darker at high concentrations.

The PEI-stabilized nanoparticles were prepared by conventional heating of aqueous solutions, adapting previous protocols from our group. For the gold nanoparticles –Au(PEI)NPs– [26,28,29,36]: water solutions of hyperbranched PEI (2 wt.%) and HAuCl_4_ (2 mM) were mixed in a 1:10 volume ratio, heated at 100 °C for 5 min under magnetic stirring, and then cooled down. For the quantum dots –CdS(PEI)QDs– [37]: an aqueous solution of hyperbranched PEI (1 wt.%) was cooled under magnetic stirring below 10 °C, and then solutions of CdCl_2_ followed by (NH_4_)_2_S (2 mM, 2:1 Vol. ratio from the previous PEI solution each) were incorporated dropwise with equivalent sequential steps, keeping the stirring for further 10 min. The resulting aqueous solutions of Au(PEI)NPs (Figure S3) or CdS(PEI)QDs (Figure S4) can be kept in a refrigerator for several weeks.

For the formation of biphasic Winsor type II mixtures, reported in preliminary studies [28,29], defined quasi-ternary mixtures (Figures S5–S7) were prepared. Dispersions of defined compositions were formed by dissolving first the AOT in the organic solvents of the oil phase, followed by adding the aqueous content and mixing at room temperature with a Vortex vibro-mixer at 2500 rpm. The purity of AOT (dry wt.%) was determined with a Sartorius moisture analyzer MA 30 (Göttingen, Germany) so that its intrinsic water was considered in the quasi-ternary compositions. The oil phase consisted of heptane with/without pentanol in a defined mass ratio, with the presence of oil-dispersible nanoparticles—0.5 wt.% SPIO(Ol)NPs and/or Ag(Ol)NPs, being the combination of SPIO(Ol)NP and Ag(Ol)NP in a 1:1 ratio—. The water phase was with the presence or absence of PEI-coated nanoparticles—0.3 wt.% Au(PEI)NPs or CdS(PEI)QDs—. The formation of nanostructured thin films resulted from drop-casting and subsequent solvent evaporation of the upper liquid phase of the biphasic Winsor type II mixtures on a given surface under variable conditions. Additionally, spin-coating (4000 rpm, 90 s) of the microemulsion dispersion was performed under vacuum with a Laurell WS-650-23 spin coater (Lansdale, PA, USA). Solvent evaporation was allowed at ambient conditions overnight for all cases.

### 2.2. Characterization Methods

The nanostructures in the resulting films formed on carbon-coated grids of copper were visualized by transmission electron microscopy (TEM) using a JEOL JEM-1011 microscope (Tokyo, Japan) at an acceleration voltage of 80 kV. The equivalent films formed in silicon wafers were visualized by scanning electron microscopy (SEM) using a Hitachi S-4800 microscope (Tokyo, Japan) at an acceleration voltage of 2 kV. UV–Vis transmittance spectra were recorded with a Shimadzu UV-2600 spectrophotometer (Kyoto, Japan) in the wavelength range between 300 and 1300 nm, using glass slides or the homologous quartz surfaces, for the transmittance of the resulting films.

An overview of the compositions of the prepared nanostructured films, as well as their respective characterization and study, can be found in Table S1.

## 3. Results

To adapt the phase properties of microemulsion dispersions, quasi-ternary mixtures of oil phase, water phase, and surfactant in defined proportions were prepared [29]. For this case, phase separation occurs with a major proportion of the oil phase solvents, in which the minor concentration of AOT surfactant cannot incorporate further water in a single isotropic, reverse microemulsion phase (Figure S5). Thus, a biphasic Winsor type II mixture is formed, consisting of an upper water-in-oil phase and a lower aqueous phase. Further addition of water will increase the volume fraction of the lower phase as excess water. The upper liquid phase, associated with water-in-oil microemulsions, had a characteristic glimmering effect whose color was provided by the oleyl-capped nanoparticles. As previously reported, the surface assembly of oil-dispersible SPIO(Ol)NPs and water-dispersible Au(PEI)NPs in filament networks is formed by drop-casting and solvent evaporation of the upper reverse microemulsion phase on a given surface. The subsequent assembly was previously interpreted based on controlled clustering of nanoparticles and the elongation of microemulsion droplets. The resulting nanostructures were mostly composed of the SPIO(Ol)NPs, if then a minor proportion of Au(PEI)NPs was necessary to form defined patterns [28,29].

For a better understanding of the scope and possibilities of these assemblies, different conditions were tested (Table S1). Firstly, the formation of these assemblies can occur over different types of surfaces under different conditions. On the one hand, different solvents in the oil phase formed different assemblies, related to different phase behavior. On the other hand, it was possible to form equivalent assemblies with oleyl-capped nanoparticles and PEI-coated nanoparticles made of different composition materials in different nanoparticle combinations.

### 3.1. Effect of the Deposition Conditions During the Surface Assembly

**Figure 1:**
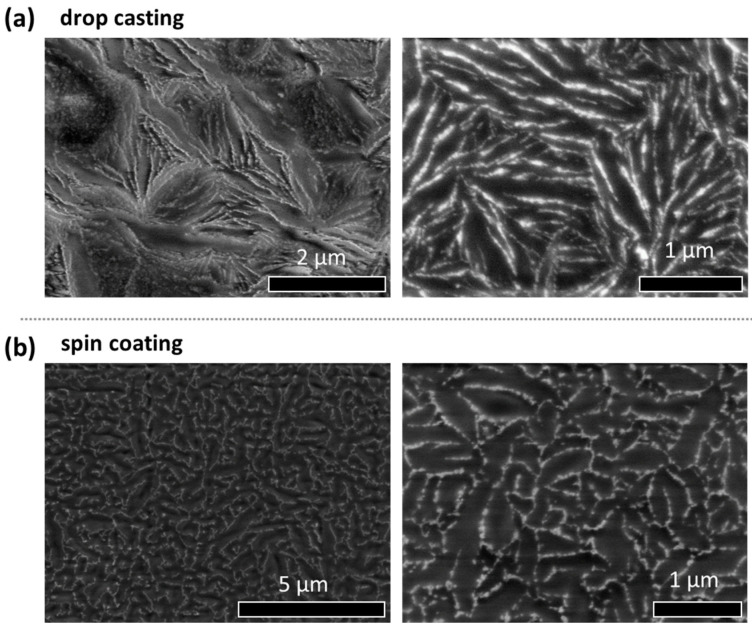
SEM at different scales of nanostructured assembled films over the surfaces of silicon wafers, using different deposition techniques of the upper AOT microemulsion phase, at 30 wt.% aq. content, containing heptane–pentanol as oil solvents: (**a**) drop-casting; (**b**) spin-coating.

By simple drop-casting of the upper phase of the Winsor type II mixture on TEM grids, the formation of filament networks from the nanoparticles in the microemulsion phase was firstly revealed [28,29]. Another previous characterization was performed on the resulting films over microscope glass slides, revealing optical properties related to the nanoparticle arrangements [29]. To confirm the formation of the same assembly in filament networks over a different surface, silicon wafers were also used to visualize the nanostructured films after solvent evaporation. Different processes were tested for the nanoparticle surface assembly. In addition to drop-casting, spin-coating was also used to form a more homogeneous film for a homogeneous spreading over the silicon wafer. As visualized by SEM (Figure 1), filament networks were also formed by drop-casting over the silicon wafer (Figure 1a), analogously to the morphology of the assembled nanostructures on a TEM grid. On the other hand, the resulting nanostructures by spin-coating showed also a network-like morphology of more regular shapes, if then the nanoparticle organizations were slightly distorted from filaments, producing shorter and more homogeneous extensions (Figure 1b). In any case, the formation of regular nanostructures could be proven over different surfaces and at different evaporation conditions (Table S1).

### 3.2. Effect of the Oil Solvents in the Microemulsion Phase

Given a fixed oil-to-surfactant ratio in the Winsor type II mixtures, it was previously found that better-defined structures are formed within a defined range of water contents after an optimal time for phase equilibration [28]. Additionally, the type of solvents can also influence the optimal time for phase separation and equilibration [28]. The influence of oil solvents on the properties of water-in-oil microemulsions is based on the interactions of the hydrocarbon solvent chains with the surfactant tails, as well as the interpenetration in the water–oil interface [38,39,40]. For further insight, the effect of pentanol as a co-surfactant in the formation of reverse microemulsions was studied by comparing the microemulsion phase properties, using heptane–pentanol or only heptane in the oil phase. As previously reported, pentanol was enhancing the flexibility of the surfactant film. For instance, pentanol was found to facilitate the incorporation of water and the extension of the isotropic phase region in the ternary diagrams (Figures S5 and S6), and its visualization by cryo–SEM showed the formation of smaller microemulsion droplets and the transition to bicontinuous phases [24,29]. Moreover, pentanol itself as a solvent in the oil phase should also contribute to the elongation of water droplets [24].

**Figure 2:**
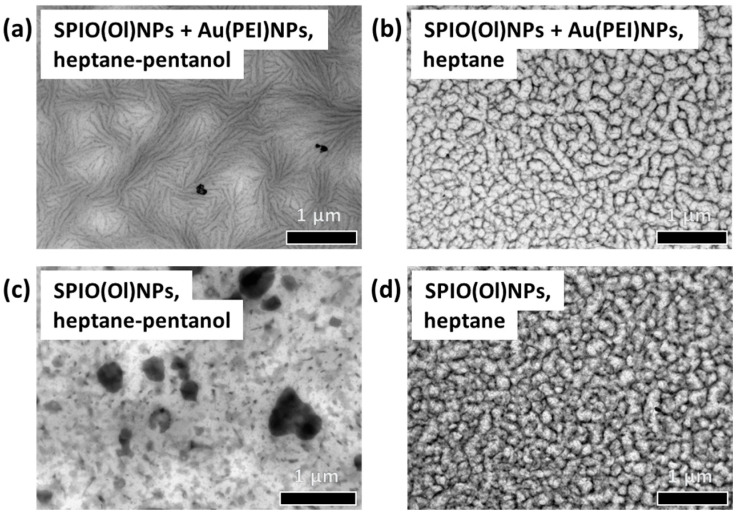
TEM of surface-assembled nanostructured films by drop-casting over grids of the upper AOT microemulsion phase, at 30 wt.% aq. content, with different combinations of nanoparticles and oil solvents: (**a**) SPIO(Ol)NPs and Au(PEI)NPs in heptane–pentanol; (**b**) both SPIO(Ol)NPs and Au(PEI)NPs in heptane; (**c**) only SPIO(Ol)NPs in heptane–pentanol; and (**d**) only SPIO(Ol)NPs in heptane.

The effect of pentanol on the nanoparticle assembly was studied by comparing the nanoparticle assemblies from the upper microemulsion phase of the biphasic Winsor type II mixture, using heptane–pentanol or with only heptane in the oil phase (Table S1). As visualized by TEM, pentanol seems to induce also a shaping effect on the morphology of the resulting nanostructures (Figure 2). When using only heptane, honeycomb-like nanostructures were observed instead of the filaments network in the presence of pentanol (Figure 2b,d). Additionally, the formation of similar honeycomb-like nanostructures is also possible with only oil-dispersible nanoparticles (Figure 2d). This also differs from the case of Winsor type II phases with heptane–pentanol as the oil phase, in which both oil-dispersible and PEI-coated nanoparticles were necessary to form the filament networks (Figure 2a). Thus, both pentanol and PEI-coated nanoparticles were required to form elongated structures, while heptane alone enables the use of only oil-dispersible nanoparticles.

### 3.3. Effect of the Type of Nanoparticle Material Composition and Their Combinations

Other types of nanoparticles can also be incorporated in the quasi-ternary mixtures while keeping similar oleyl ligands and PEI for the oil and aqueous phases, respectively (Figure S7). The upper reverse microemulsion phase had a characteristic color provided by the oleyl-capped nanoparticles: brown for SPIO(Ol)NPs and yellow for Ag(Ol)NPs. The lower liquid phase, with the excess of water that could not be incorporated in the microemulsions, had a characteristic color from the destabilized PEI-coated nanoparticles, being violet for Au(PEI)NPs or white for CdS(PEI)QDs. The resulting hybrid nanostructured films, after drop-casting of the upper water-in-oil phase in the presence of different nanoparticle combinations, presented similar nanostructures with tunable morphologies and light absorption properties (Table S1).

**Figure 3:**
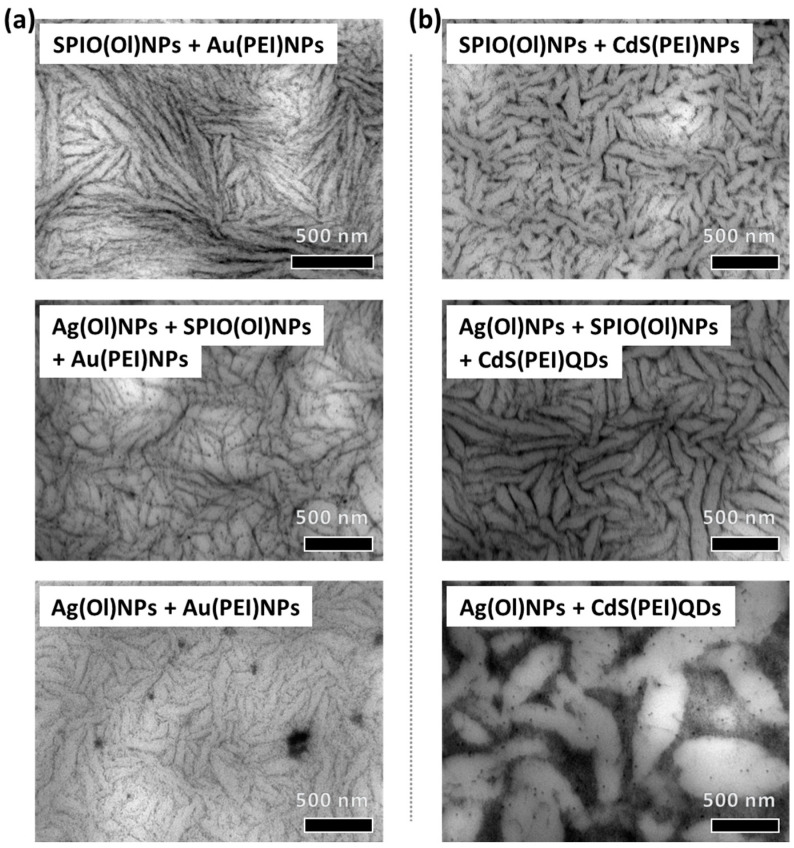
TEM of surface-assembled nanostructured films by drop-casting over grids of the upper microemulsion phase at 30 wt.% aq. content, containing heptane–pentanol as oil solvents, with different nanoparticle combinations: (**a**) films formed with SPIO(Ol)NPs and/or Ag(Ol)NPs from the oil phase and with Au(PEI)NPs from the water phase; (**b**) films formed with SPIO(Ol)NPs and/or Ag(Ol)NPs from the oil phase and with CdS(PEI)QDs from the water phase.

**Figure 4:**
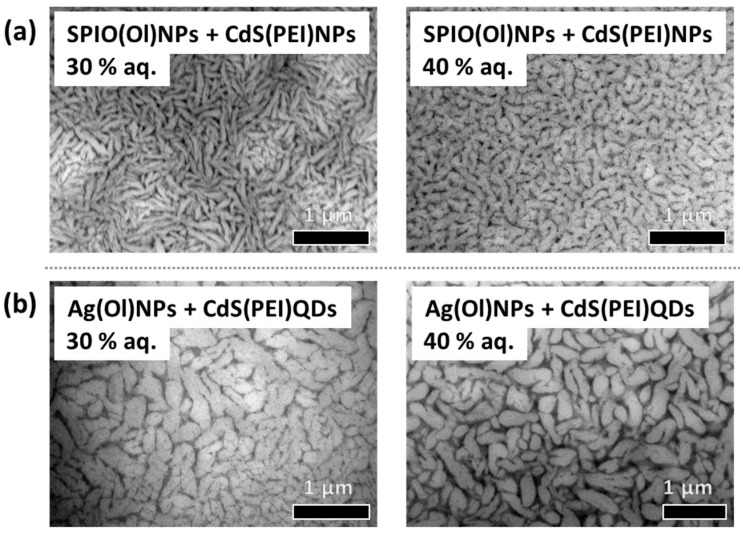
TEM of surface-assembled nanostructured films by drop-casting over grids of the upper AOT microemulsion phase at 30 wt.% (left) or 40 wt.% (right) aq. content, containing heptane–pentanol as oil solvents, in the presence of different nanoparticle combinations: (**a**) films formed with SPIO(Ol)NPs and CdS(PEI)QDs; (**b**) films formed with Al(Ol)NPs and CdS(PEI)QDs.

Visualization by TEM could prove that the filament networks can also be formed from dispersions with other nanoparticle compositions by keeping equivalent surface functionalization (Figure 3). When replacing SPIO(Ol)NPs with Ag(Ol)NPs in the presence of Au(PEI)NPs, thinner filaments are formed and additional agglomerates appear around (Figure 3a). It is also possible to form such nanostructures with a mixture of both SPIO(Ol)NPs and Ag(Ol)NPs, resulting in intermediate nanostructured morphologies. By replacing the aqueous Au(PEI)NPs with CdS(PEI)QDs, significantly thicker filaments of shorter lengths were formed (Figure 3b). The thickness of these filaments in the presence of CdS(PEI)QDs also fluctuates at different areas of the assembly extension, in a wave-like trend. For the case of oil-dispersible SPIO(Ol)NPs, a transition towards shorter filaments within more cross-points occurs by increasing aquous content of the initial mixure (Figure 4). These morphologies were previously reported for a broader range of water content in the presence of Au(PEI)NPs [28], and a similar behavior is also observed in the presence of CdS(PEI)QDs (Figure 4a). This shortening of the filaments is less significant at higher aqueous contents for the case of Ag(Ol)NPs as oil-dispersible nanoparticles and CdS(PEI)QDs from the aqueous phase (Figure 3b and Figure 4b), if then more abundant cross-points between filaments are still observed over a given area.

**Figure 5:**
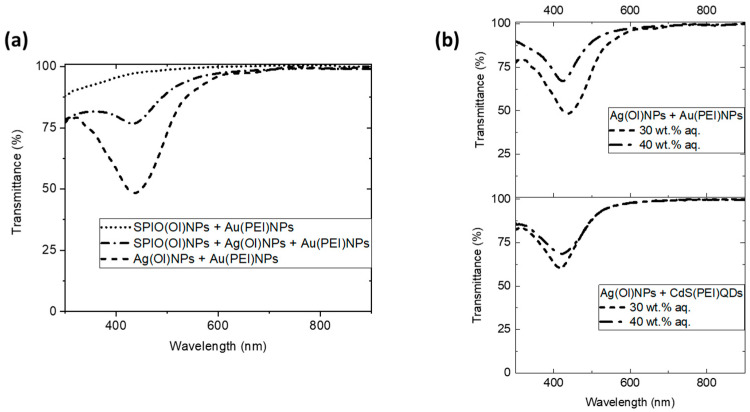
UV–Vis transmittance of surface-assembled nanostructured films by drop-casting over glass slides of the upper AOT microemulsion phase, containing heptane–pentanol as oil solvents, with different nanoparticle combinations and water contents: (**a**) films formed at 30 wt.% aq. content with SPIO(Ol)NPs and/or Ag(Ol)NPs from the oil phase and with Au(PEI)NPs from the water phase; (**b**) films formed at 30 wt.% or 40 wt.% aq. content, with Ag(Ol)NPs from the oil phase and with Au(PEI)NPs (top) or CdS(PEI)QDs (bottom) from the water phase.

Regarding the optical properties of these nanoparticle assemblies, previous characterization by UV–Vis absorption on the upper microemulsion phase (before drop-casting) indicated a major presence of the oil-dispersible SPIO(Ol)NPs if then the characteristic plasmon band of the Au(PEI)NPs could also be observed and tuned at different quasi-ternary compositions and equilibration times [28]. Besides, previous UV–Vis transmittance on the resulting transparent nanostructured films (after drop-casting) showed a major presence of SPIO(Ol)NPs, by which the absorption profile of the Au(PEI)NPs (Figure S3c) is not distinguishable [29]. Thus, the transmittance profiles of these transparent films (Figure 5) only reveal the absorption bands of the oleyl-capped nanoparticles. The absorption peaks observed on the nanoparticle-assembled films could reveal the red-shifted plasmon peak of the Ag(Ol)NPs (Figure S2c), of tuneable intensity when Ag(Ol)NPs are combined with SPIO(Ol)NPs in the oil phase (Figure 5a). Similar transmittance behaviour of nanostructured films in the major presence of Ag(Ol)NPs was also observed in the presence of either Au(PEI)NPs or CdS(PEI)QDs in the aqueous phase (Figure 5b). Moreover, the characteristic absorption band of Ag(Ol)NPs was reduced at higher contents of the aqueous phase (Figure 5b), indicating that a higher ratio of the lower aqueous phase in the Winsor type II mixture (Figures S5 and S7) likely induces diffusion or destabilization of the Ag(Ol)NPs from the upper microemulsion phase to the lower aqueous phase. Therefore, the increase of aqueous content in the phase-separated mixtures would not increase the concentration of Ag(Ol)NPs in the oil phase.

## 4. Discussion

The formation of these nanostructures from quasi-ternary microemulsion systems is proven to be tunable under different conditions (Table S1). At a fixed oil-to-surfactant ratio (Figure S5), an optimal range of water content in the biphasic Winsor Phase mixture was different for each combination of oil solvent and nanoparticle components. The overall process was previously interpreted according to a controlled clustering of nanoparticles, triggered by the charge suppression of PEI on their surface as well as the elongation of the water-in-oil microemulsion droplets [28]. Moreover, based on previous findings, too little aqueous content results in insufficient partitioning and distribution of nanoparticles to provide well-defined nanostructures, while excessive incorporation of water results in uncontrolled or unordered agglomeration of nanoparticles [28]. Within the optimal range of water content (Figure S5), it was also found that the further incorporation of water contributed to a shorter filament length in a better-packed network of filaments with more abundant cross-points [28]. It would be of further interest to achieve the formation of these filament-like networks from a single microemulsion phase, so that phase separation does not have to occur previously. At the same oil-to-surfactant mass ratio (95:5) as for the biphasic mixtures (Figure S5), possible nanoparticle assemblies from drop-casting of single-phase microemulsions, without excess water causing phase separation, were not possible to visualize by TEM. This is attributed to a hampered transmission of electrons, which should be likely due to an excess of AOT remaining in the resulting films. Thus, lower surfactant concentrations could be further investigated to provide equivalent single-phase microemulsions for the upper phase of a biphasic mixture.

Based on the present findings, the morphology of the nanostructured assemblies could be reproduced by drop-casting over different types of surfaces (Figure 1, Figure 2, Figure 3 and Figure 4). Additionally, the morphology of these assemblies can be tuned by using spin-coating, and more regular arrangements can be obtained (Figure 1b). Further insights about the nanoparticle organization and thin film properties could be provided by applying different spinning rates and evaporation conditions, such as temperature and vapor pressure [41]. Although the assembly is formed by solvent evaporation over a surface, certain 3D organization might be involved in the resulting films. It is likely that the nanoparticle organization might also be conditioned by the presence of a surrounding gel-like matrix of remaining AOT and water. Similar nanostructures were obtained by surfactant aggregates over a surface [27]. Interestingly, liquid crystalline mesophases would still be present (Figure S5) [29,31] and could also lead to interesting optical properties [29,42,43]. Therefore, the resulting optoelectronic properties could be investigated in more detail.

Additionally, the formation of ordered arrangements was observed in the presence of different organic solvents in the oil phase. The anisotropic, elongated shapes of the resulting assembly from spherical and small nanoparticles should be attributed to adsorption-related effects of nanoparticles at liquid–liquid interfaces [44]. Yet, the presence of pentanol in the oil phase is also relevant for the formation of filament networks (Figure 2). Thus, the formation of filament networks required the presence of both oleyl-capped and PEI-coated nanoparticles, as well as a mixture of pentanol with a nonpolar oil solvent (Figure 2a) [28,29]. On the other hand, more regular honeycomb-like nanostructures were formed from dispersions without pentanol, which can also be formed in the absence of the PEI-coated nanoparticles (Figure 2b,d). Noteworthy, the presence of alkanol in the continuous oil phase also plays a role in the elongation of water-in-oil microemulsion droplets [24,28]. The composition of the oil solvents in the continuous phase has also an effect on the shape and properties of the surfactant film in the microemulsion droplets [24], besides the nanoparticle interactions at the liquid–liquid interface [29]. Based on the co-surfactant effect of middle-chain alcohols, the increase of the interfacial area of the surfactant film would change the sizes and shapes of microemulsions [21]. On the other hand, the type of oil solvents can also influence the optimal time of phase equilibration to form regular nanostructures. For instance, it was previously reported that several days are required to obtain better ordering of the assemblies when using hexane–pentanol as the oil phase, while dispersions with heptane–pentanol as the oil phase required only one day of phase separation and resulted in disordered arrangements for further equilibration days [28]. Therefore, both conditions of water content and phase equilibration time for optimal assemblies depend on the type of solvents used in the oil phase. The polarity of the solvents present has also an influence on the self-organization of AOT dispersions, as well as the interfacial tension by co-surfactant effects [20,45]. This should conditionate the morphology of the assembled nanostructures, as the solvent evaporation would force the packing of the self-organized constituents from the initial dispersion. Thus, the remaining surfactant structures should be based on closed-packed arrangements, in similarity with liquid crystalline mesophases [29,31].

It was also demonstrated that the nanostructured assemblies can be constituted by other nanoparticles of different material compositions, if then their surface functionalization should be preserved (Figure 3 and Figure 4). The hydrophobic chains for the oil-dispersible nanoparticles included oleyl derivatives [32,33,34,35,46], while PEI as a polycation was used to coat the water-dispersible nanoparticles [36,37]. The monodispersity of the oil-dispersible nanoparticles (Figures S1b and S2b), provided by the diol and oleyl ligands, should play a relevant role in the periodicity of these arrangements [29,46,47]. Periodic inter-particle distances should be attributed to the interdigitation of oleyl ligands within the monodisperse oil-dispersible nanoparticles, contributing substantially to the formation of these well-ordered arrangements [29,46]. On the other hand, the PEI-coating has been proven to interact strongly with the AOT interfacial layer [29,48], so that controlled aggregation is enabled. Other possibilities of polyelectrolyte coating could also be explored for self-assembly processes in microemulsions. This would include the interaction with other surfactants with different head groups or the influence of the pH and salinity conditions.

Similarly, it was also proven the formation of nanoparticle filament networks by incorporating different nanoparticle combinations, including mixtures of different oleyl-capped nanoparticles (Figure 3). Although the nanoparticle materials can be of different compositions, other effects might occur depending on surfactant interactions at their surface. For instance, Ag(Ol)NPs might tend to interact more strongly with AOT (Figure S7b) compared to SPIO(Ol)NPs (Figure S6b). In any case, the aqueous PEI-coated nanoparticles might compensate for oleyl-capped nanoparticle interactions at the water–oil interface (Figure S7). Additionally, the morphology of the resulting nanostructures also changes according to the aqueous content of the self-organized dispersion (Figure 4). The filament length of filament networks tends to get shortened upon increasing aqueous content if shorter filaments can also be obtained at the same aqueous content for different nanoparticle compositions (Figure 3 and Figure 4). Thus, an optimal content of the aqueous phase should be characteristic of each combination of nanoparticles and organic solvents.

Controlling the aggregation and interaction effects of nanoparticles in these surfactant-stabilized liquid dispersions is a key feature for the formation of organized assemblies [26,28]. The confined clustering of PEI-stabilized nanoparticles with AOT, forming polyelectrolyte–surfactant complexes by electrostatic interactions, plays an important role in the formation of the filament networks [28,29,48]. In any case, the use of only oleyl-capped nanoparticles to form ordered arrangements in the presence of only non-polar solvents as the oil phase is also demonstrated (Figure 2). Other strategies of partial nanoparticle destabilization might be applied to trigger the controlled clustering effect.

Potential applications of these nanostructured films could be based on their polarized transmittance or in the combination of properties of nanoparticles or liquid phases, including different nanomaterials as hybrid nanostructures (Figure 5). For instance, the collective plasmonic properties of nanoparticles can be used for surface catalysis or sensing [1,2,11,30,49]. Other optoelectronic properties could be provided by metal oxides and semiconductor materials, as well as combinations in hybrid nanomaterials [1,11]. Applications based on micropatterning are related to sensing and microelectronics [1,2,7,8,10,50]. However, contact with other liquid solutions in the present nanostructures might degrade or disassemble the nanoparticles, so that either a reproducible fixation technique is needed or gaseous species would be preferred to be used for their surface interactions. Some possibilities to fix these assembled nanostructures include the complete drying of the remaining water in the nanostructured films, controlled sintering of the nanoparticles, or the vapor deposition of other coating species. A different approach could involve the use of non-aqueous solvents to substitute the water phase so that additional conductive properties could be provided. Further research that explores tunable properties is needed for the targeted use of these hybrid nanostructures.

## 5. Conclusions

The formation of nanoparticle-assembled films from water-in-oil dispersions with AOT as a surfactant was demonstrated for quasi-ternary mixtures of different compositions and for different conditions during solvent evaporation. This versatility included similar morphologies of nanostructured assemblies over different surfaces, different deposition conditions, as well as different combinations of nanoparticles and oil solvents, having each mixture a variable range of optimal aqueous contents.

Firstly, the resulting nanoparticle assemblies were proven to be formed over different surfaces, as equivalent morphologies were observed on grids or silicon wafers as substrates. By using different deposition processes over silicon wafers (drop-casting or spin-coating), the morphology of the assemblies can also be tuned.

The nanoparticle films could also be assembled using different oil solvents in the quasi-ternary mixtures. The change in morphology, from filament networks using heptane–pentanol to honeycomb-like arrangements using only heptane, proved a tuning effect of middle-chain alcohols on the formation of nanoparticle filaments. This is likely due to a microemulsion droplet elongation related to the co-surfactant stabilization of the water–oil interface. Both oleyl- and PEI-coated nanoparticles were required to form ordered arrangements in the presence of pentanol. Besides, ordered assemblies of only oleyl-capped nanoparticles can be provided with only heptane as an oil solvent.

By incorporating different combinations of nanoparticles with equivalent surface coating, the morphologies of the surface-assembled nanostructures in filament networks were also equivalent. The resulting nanostructured films of different nanoparticle compositions can be tuned, indicating optimal ratios for each quasi-ternary mixture. This enables the use of different nanoparticle materials to provide tunable collective properties in the resulting nanostructured films.

Further investigations should be made, including additional characterization and the testing of further combinations of nanoparticles and solvents. Especially the collective optoelectronic properties from individual nanoparticles could prove interesting applications of these nanostructured films.

## Data Availability

The original contributions presented in this study are included in the article and Supplementary Materials. Further inquiries can be directed to the corresponding authors.

## Supplementary Materials

The following supporting information can be downloaded at: https://www.mdpi.com/article/10.3390/nano14211726/s1, Figures S1–S4: characterization of SPIO(Ol)NPs, Ag(Ol)NPs, Au(PEI)NPs, and CdS(PEI)QDs (respectively); Figures S5–S7: quasi-ternary phase diagrams showing the isotropic phase composition regions associated to water-in-oil microemulsions, with different nanoparticle combinations incorporated in the respective oil and aqueous phases, using different solvents in the oil phase; Table S1: Compositions of the nanostructured films and the used characterization methods, for the corresponding comparisons regarding the present study about their versatility.
