# Supplementary material for: Versatility of the Templated Surface Assembly of Nanoparticles from Water-in-Oil Microemulsions in Equivalent Hybrid Nanostructured Films
Source: Nanomaterials (Basel). 2024 Oct 29;14(21):1726. doi: 10.3390/nano14211726 (PMC11548002; doi:10.3390/nano14211726)
Supplement: Supplementary file 1 [file nanomaterials-14-01726-s001.zip › nanomaterials-3244682-supplementary.pdf]

Supplementary Material

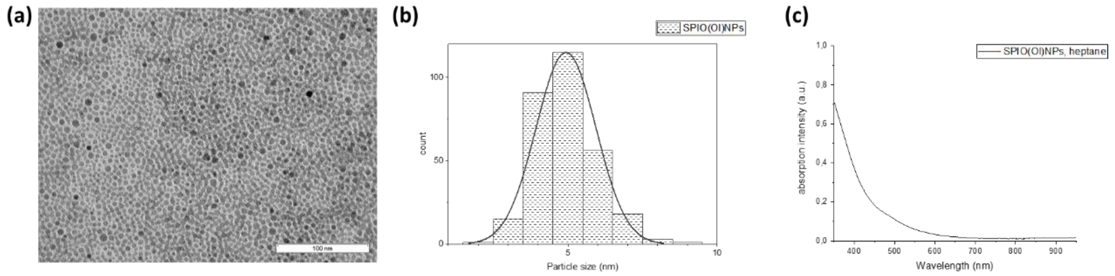

Figure S1. Characterization of SPIO(Ol)NPs: (**a**) TEM micrograph; (**b**) resulting histogram from size distribution of 300 nanoparticle counts; (**c**) UV-Vis absorption spectra of diluted dispersion.

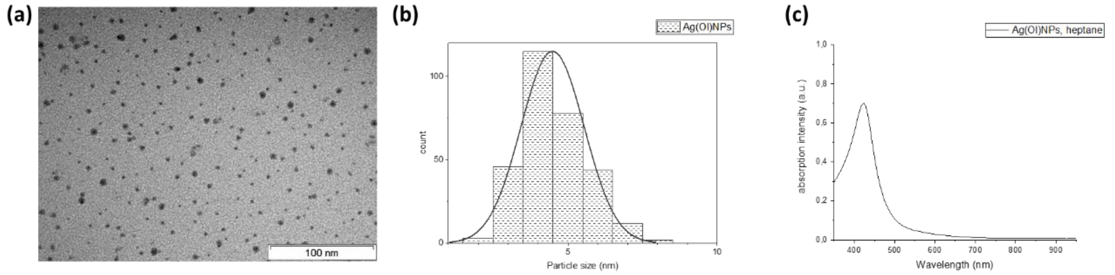

Figure S2. Characterization of Ag(Ol)NPs: (**a**) TEM micrograph; (**b**) resulting histogram from size distribution of 300 nanoparticle counts; (**c**) UV-Vis absorption spectra of diluted dispersion.

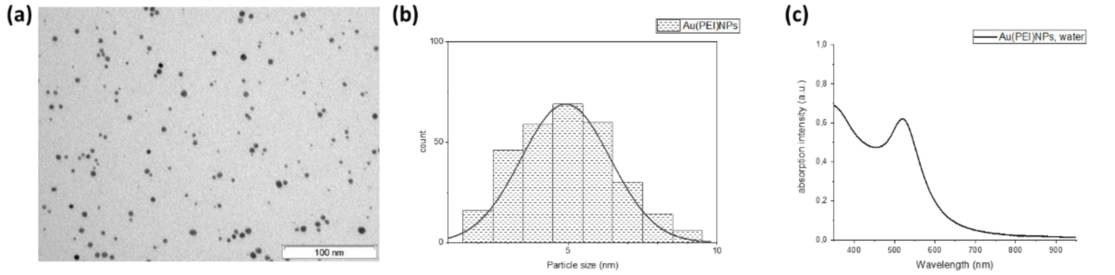

Figure S3. Characterization of Au(PEI)NPs: (**a**) TEM micrograph; (**b**) resulting histogram from size distribution of 300 nanoparticle counts; (**c**) UV-Vis absorption spectra of diluted dispersion.

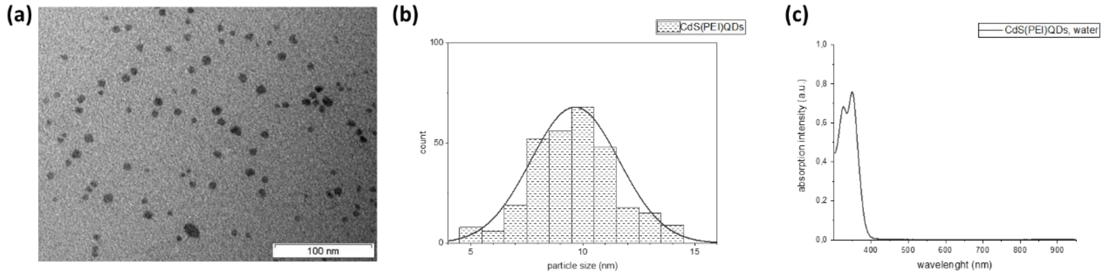

Figure S4. Characterization of CdS(PEI)QDs: (**a**) TEM micrograph; (**b**) resulting histogram from size distribution of 300 nanoparticle counts; (**c**) UV-Vis absorption spectra of diluted dispersion.

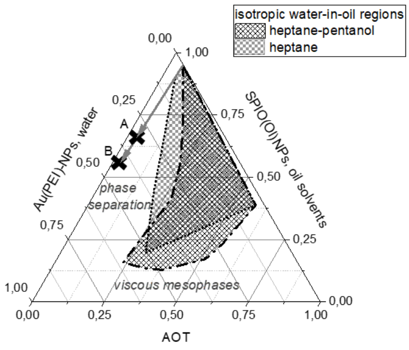

Figure S5. Quasi-ternary phase diagram showing the isotropic phase composition regions associated to water-in-oil microemulsions, with SPIO(Ol)NPs dispersed in the oil phase and Au(PEI)NPs in the aqueous phase, using different solvents in the oil phase. Composition points A and B indicate the biphasic dispersions at 30 wt.% and 40 wt.% aqueous contents, respectively, along a dilution line at oil-to-surfactant 95:5 mass ratio.

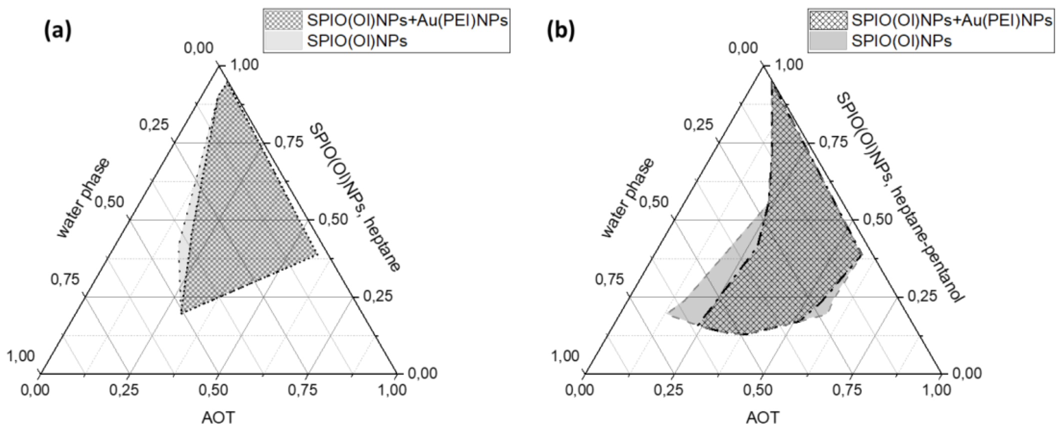

Figure S6. Quasi-ternary phase diagram showing the isotropic regions, with SPIO(Ol)NPs in the oil phase, in presence or absence of Au(PEI)NPs in the aqueous phase: (**a**) using heptane as oil phase; (**b**) using heptane-pentanol as oil phase.

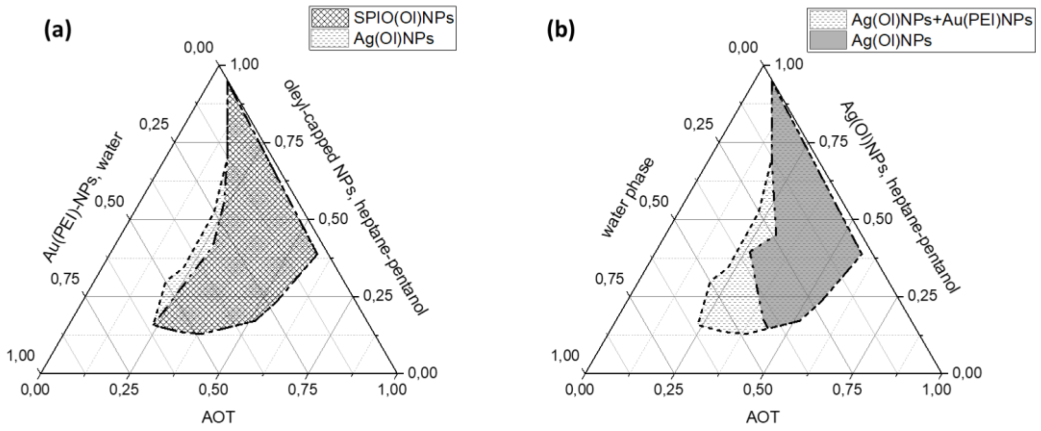

Figure S7. Quasi-ternary phase diagram showing the isotropic regions, with different nanoparticle combinations, using heptane-pentanol as oil phase: (**a**) SPIO(Ol)NPs or Ag(Ol)NPs in presence of Au(PEI)NPs; (**b**) Ag(Ol)NPs in presence or absence of Au(PEI)NPs.

Table S1. Compositions of the nanostructured films and the used characterization methods, for the corresponding comparisons regarding the present study about their versatility.

Oil solvents	Incorporated nanoparticles	Aq. content	Characterization methods
heptane-pentanol	SPIO(Ol)NPs and Au(PEI)NPs	30 wt. %	SEM[1], TEM[1,2,3], UV-Vis transmittance[3]
heptane-pentanol	SPIO(Ol)NPs	30 wt. %	TEM[2]
heptane	SPIO(Ol)NPs and Au(PEI)NPs	30 wt. %	TEM[2]
heptane	SPIO(Ol)NPs	30 wt. %	TEM[2]
heptane-pentanol	Ag(Ol)NPs and Au(PEI)NPs	30, 40 wt. %	TEM[3], UV-Vis transmittance[3,4]
heptane-pentanol	SPIO(Ol)NPs, Ag(Ol)NPs and Au(PEI)NPs	30 wt. %	TEM[3], UV-Vis transmittance[3]
heptane-pentanol	SPIO(Ol)NPs and CdS(PEI)QDs	30, 40 wt. %	TEM[3,4]
heptane-pentanol	Ag(Ol)NPs and CdS(PEI)QDs	30, 40 wt. %	TEM[3,4], UV-Vis transmittance[3,4]
heptane-pentanol	SPIO(Ol)NPs, Ag(Ol)NPs and CdS(PEI)QDs	30 wt. %	TEM[3]

[1] Comparison regarding different surfaces and deposition methods.
[2] Comparison regarding different composition of solvents in the oil phase.
[3] Comparison regarding different combinations of nanoparticles.
[4] Comparison regarding different aqueous content.
